# Division within the North American boreal forest: Ecological niche divergence between the Bicknell's Thrush (*Catharus bicknelli*) and Gray‐cheeked Thrush (*C. minimus*)

**DOI:** 10.1002/ece3.3080

**Published:** 2017-06-08

**Authors:** Alyssa M. FitzGerald

**Affiliations:** ^1^ Department of Biological Sciences University at Albany Albany NY USA; ^2^ New York State Museum 3140 Cultural Education Center Albany NY USA

**Keywords:** boreal forest, *Catharus* thrushes, ecological niche divergence, ecological speciation, niche modeling, sister species

## Abstract

Sister species that diverged in allopatry in similar environments are expected to exhibit niche conservatism. Using ecological niche modeling and a multivariate analysis of climate and habitat data, I test the hypothesis that the Bicknell's Thrush (*Catharus bicknelli*) and Gray‐cheeked Thrush (*C. mimimus*), sister species that breed in the North American boreal forest, show niche conservatism. Three tree species that are important components of breeding territories of both thrush species were combined with climatic variables to create niche models consisting of abiotic and biotic components. Abiotic‐only, abiotic+biotic, and biotic‐only models were evaluated using the area under the curve (AUC) criterion. Abiotic+biotic models had higher AUC scores and did not over‐project thrush distributions compared to abiotic‐only or biotic‐only models. From the abiotic+biotic models, I tested for niche conservatism or divergence by accounting for the differences in the availability of niche components by calculating (1) niche overlap from ecological niche models and (2) mean niche differences of environmental values at occurrence points. Niche background similarity tests revealed significant niche divergence in 10 of 12 comparisons, and multivariate tests revealed niche divergence along 2 of 3 niche axes. The Bicknell's Thrush breeds in warmer and wetter regions with a high abundance of balsam fir (*Abies balsamea*), whereas Gray‐cheeked Thrush often co‐occurs with black spruce (*Picea mariana*). Niche divergence, rather than conservatism, was the predominant pattern for these species, suggesting that ecological divergence has played a role in the speciation of the Bicknell's Thrush and Gray‐cheeked Thrush. Furthermore, because niche models were improved by the incorporation of biotic variables, this study validates the inclusion of relevant biotic factors in ecological niche modeling to increase model accuracy.

## 1 INTRODUCTION

Whether speciation results in niches that are conserved or divergent remains a highly contested question within evolutionary biology. Sister taxa show niche conservatism if niche characteristics were ancestrally inherited and if speciation occurs in similar environments with no ecological selection (Peterson, Soberón, & Sánchez‐Cordero, 1999; Warren, Glor, & Turelli, 2008; Wiens & Graham, 2005; Wooten & Gibbs, 2012). For example, Peterson et al. (1999) showed that most of 37 sister taxon pairs of birds, mammals, and butterflies in southern Mexico had similar ecological characteristics, but ecological niches were less similar at the familial level. Similarly, closely related North American wood‐warblers (*Parulidae*) are more likely to have similar niches than more genetically distant warbler pairs (Lovette & Hochachka, 2006). McCormack, Zellmer, and Knowles (2009) found that although allopatric Mexican Jay (*Aphelocoma ultramarina*) subspecies show niche differences, these dissimilarities are due to differences in available conditions and not niche divergence. These studies, among others, concluded that sister taxa predominantly reside in niches that are conserved over time, and that any minor niche differences tend to accrue after allopatric speciation. Adaptation to different environments, on the other hand, results in ecological niche divergence between taxa (Hendry, 2009; Schluter, 2009), and studies that find evidence for niche divergence infer that adaptation was a strong component of speciation. For example, *Sistrurus* rattlesnake subspecies generally showed niche divergence, and recently diverged subspecies were more likely to show niche divergence than more distantly related subspecies (Wooten & Gibbs, 2012). Despite ongoing gene flow after secondary contact, parrotbills (*Paradoxornis*) in eastern Asia show significant niche differentiation in allopatry and sympatry, implying that ecological selection is to maintain the species’ separation (Shaner et al., 2015). Sister taxa that reside in non‐identical niches and show corresponding genetic or phenotypic breaks, such as Sage Sparrow subspecies (*Artemisiopiza belli*; Cicero & Koo, 2012), skinks in the *Plestiodon skiltonianus* species complex (Wogan & Richmond, 2015), or chimpanzee (*Pan troglodytes*) subspecies (Sesink‐Clee et al., 2015), are often cited as showing niche divergence.

Disagreement on whether closely related taxa are more likely to exhibit niche conservatism or divergence often stems from testing different methodologies and hypotheses (Warren et al., 2008). Many studies published prior to 2008 inferred ecological speciation if sister taxa showed any adaptive, behavioral, or ecological differences (Hendry, 2009), but taxa will always show some degree of niche differentiation due to differences in background niche availability, such as access to different resources or environments (Rundell & Price, 2009; Warren et al., 2008; Wiens & Graham, 2005). To test for ecological divergence, it is therefore necessary to show (1) taxa are ecologically distinct and (2) these differences are not due to differences in background niche availability. Recent advances in climate data accessibility and modeling techniques allow us to predict and visualize a species’ realized (actual) and fundamental (background) niche (Hutchinson, 1957; Peterson et al., 1999) in a geographic information system framework. By creating a model from occurrence points and environmental factors, we can calculate actual and background niche differences between taxa. If actual niches are more similar than expected given the fundamental environmental differences between backgrounds, we infer that the species exhibit niche conservatism and that speciation did not occur because of ecological divergence (Anderson, Peterson, & Gόmez‐Laverde, 2002; McCormack et al., 2009; Schluter, 2009; Warren et al., 2008).

The genus *Catharus*, long recognized as a model system for examining speciation, migration, and morphological, vocal, and behavioral differentiation (Dilger, 1956; Marshall, 2001; Noon, 1981; Ouellet, 1993; Ruegg, 2007; Ruegg, Slabberkoorn, Clegg, & Smith, 2006; Voelker, Bowie, & Klicka, 2013; Winker, 2010; Winker & Pruett, 2006), consists of 12 New World species. The most recently diverged lineage comprises the threatened Bicknell's Thrush [*Catharus bicknelli* (Ridgeway, 1882)] and Gray‐cheeked Thrush [*C. minimus* (Lafresnaye, 1848)] (Voelker et al., 2013), both of which breed in the North American boreal forest (Marshall, 2001; Ouellet, 1993). These taxa were considered conspecific for more than a century (Monroe et al., 1995), and are nearly indistinguishable except by slight vocal and morphologic differences (Marshall, 2001; Ouellet, 1993). The Bicknell's Thrush breeds in New York, New England, New Brunswick, Nova Scotia, and southern Quebec, whereas the Gray‐cheeked Thrush breeds from Newfoundland across the continent to Alaska and Siberia, and these species also have allopatric wintering ranges in the Neotropics. Because these are recently diverged sister species that breed in outwardly similar boreal forest habitats, they provide an opportunity to examine niche conservatism and the role of ecological selection in speciation.

**Figure 1:**
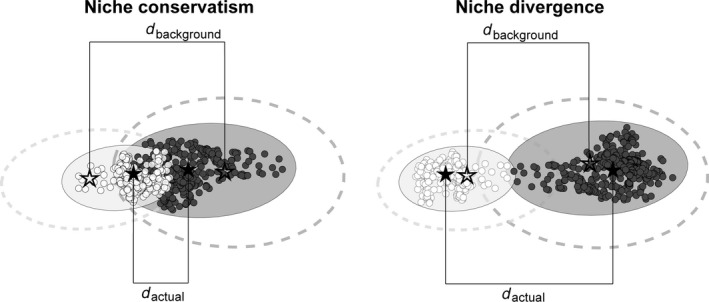
Overview of niche divergences tests for allopatric or partially overlapping species showing either niche conservatism or niche divergence. Light and dark dots are occurrences of different taxa. The filled‐in ellipses represent their actual niches (encompassing all dots) and the dotted lines represent their backgrounds, defined as the accessible area to a species. The niche background similarity test implemented in ENMT ools (Warren et al., 2008, 2010) calculates overlap between the actual niche of *A* (light filled‐in ellipse) and the background of *B* (dark dotted line), and then overlap is calculated between the actual niche of *B* (dark filled‐in ellipse) and the background of *A* (light dotted line). Niche divergence is supported when actual overlap (between the filled‐in ellipses of *A* and *B*) is significantly less than overlap between the background of one species and the actual niche of the other. The multivariate niche test adapted from McCormack et al. (2009) instead compares the differences in means of the actual niche of each species (filled‐in stars) to the means of the backgrounds (open stars). If *d*_actual_ > *d*_background_, niche divergence is supported. Note that niche overlap does not necessarily correspond with range overlap

The goal of this project is to shed light on the role of ecological selection in promoting divergence in this species complex by testing the hypothesis that the Bicknell's Thrush and Gray‐cheeked Thrush exhibit niche conservatism. I test for niche conservatism or divergence by accounting for the differences in the availability of niche components using two different methods (Figure 1). The background similarity test compares the overlap of actual and background niches based on ecological niche models (ENMs) (Warren, Glor, & Turelli, 2010; Warren et al., 2008). Because ENMs may have environmental variables that are spatially autocorrelated (McCormack et al., 2009), I also employ a multivariate analysis, which compares the difference in means of niche axes (principal components) from actual and background occurrences. A secondary goal of this project is to include biotic factors in ecological niche models to more accurately depict and categorize a species’ realized niche (de Araújo, Marcondes‐Machado, & Costa, 2014; Heikkinen, Luoto, Virkkala, Pearson, & Körber, 2007; Pearson & Dawson, 2003). Models created from abiotic‐only factors, abiotic and biotic factors, and biotic‐only factors were compared to determine the most effective variable set to use in niche divergence tests.

## 2 MATERIALS AND METHODS

### 2.1 Bird occurrence data

**Figure 2:**
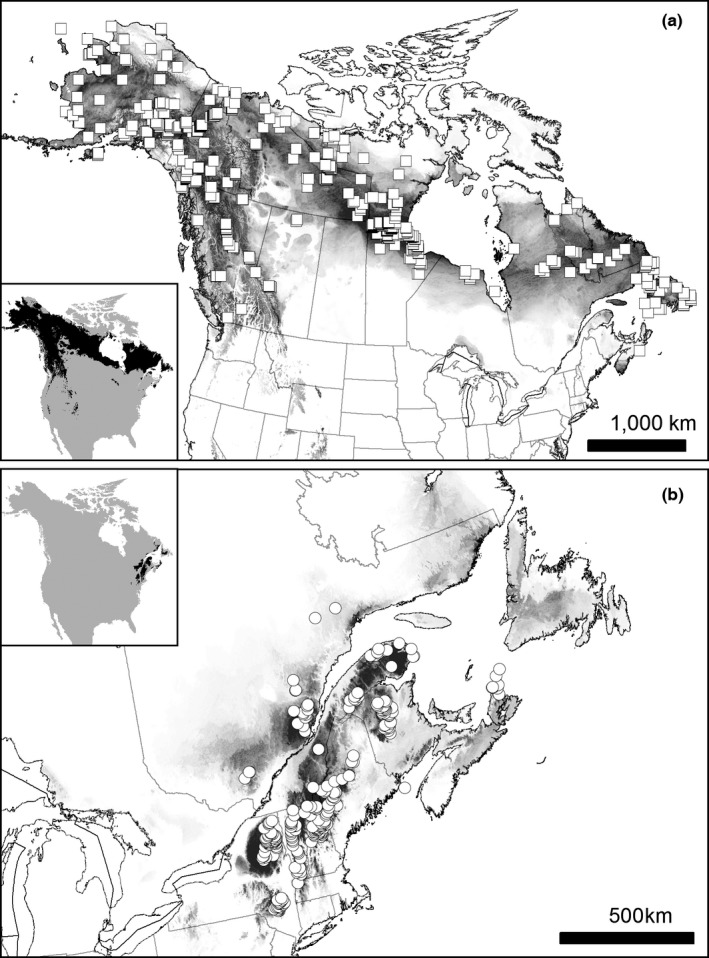
Final ecological niche models and occurrences for (a) Gray‐cheeked Thrush (squares) and (b) Bicknell's Thrush (circles) generated with climate and trees. Darker colors indicate a higher probability of occurrence. Locator maps are shown with a logistic threshold designation of 0.2

Only breeding occurrences dated from June 7 to July 31 (Bicknell's Thrush) or June 14 to July 31 (Gray‐cheeked Thrush) were included to avoid any possible migrants in passage (Kessel, 1989; Lowther, Rimmer, Kessel, Johnson, & Ellison, 2001; Rimmer, McFarland, Ellison, & Goetz, 2001). Each occurrence has a coordinate uncertainty of <5 km; if coordinates were not available, the verbal location description was georeferenced by the author. Outliers—occurrences purportedly observed beyond their currently reported ranges—were removed unless backed by strong evidence of correct identification, such as vocal recordings, museum specimens, or genotyping. Duplicates (two or more occurrences within a 5 km^2^ grid) were removed, and datasets were manually thinned to create a more uniform density across the whole range to avoid sampling bias (van Els, Cicero, & Klicka, 2012; Kramer‐Schadt et al., 2013; Phillips et al., 2009; Yackulic et al., 2013). Occurrence data were obtained from online databases of museum specimens, audio and visual recordings, standardized avian survey data, primary literature reports, and aggregated observations (see Appendix S1 in Supporting Information). Each photograph, video, and song/call recording was inspected to confirm the correct species identification. Breeding Bird Survey (BBS) stop data were examined for each route, the specific stops where thrushes were heard or seen were determined, and then stop descriptions and www.googlemaps.com were used to georeference each occurrence. Two occurrences from the literature (Lewis & Starzomski, 2015; Marshall, 2001) were included because they filled in range gaps, and both occurrences were georeferenced based on descriptions and maps in each published work. Incidental observations from eBird (www.ebird.org) were only used to supplement undersurveyed regions. Citizen scientists submit bird species sightings to eBird, and regional reviewers vet these submissions before accepting them. For these data, I examined each occurrence point, including the location, coordinate uncertainty, the other birds submitted with each checklist, the observer, and any comments posted to ensure correct species identification. I compiled 274 occurrences for Bicknell's and 534 for Gray‐cheeked Thrush (Figure 2 and Appendix S1) For both species, >25% of the occurrences are vouchered by genotyped tissue samples, specimens, or recordings; fewer than 5% of occurrences were obtained from incidental observations reported to eBird.

### 2.2 Tree species selection and occurrences

A literature review was conducted to identify tree species that are important components of breeding territories of both thrush species. I identified five studies for Bicknell's Thrush and three for Gray‐cheeked Thrush that included quantitative habitat measurements at confirmed breeding localities (Appendix S2). These studies quantified habitat in areas ranging in scale from ~330 to 4,160 m^2^, usually within a contiguous area, and were conducted in New Hampshire, Quebec, New Brunswick, Newfoundland and Labrador, and Alaska.

For Bicknell's Thrush sources (Aubry, Desrochers, & Seutin, 2011; Connolly, Seutin, Savard, & Rompré, 2002; McKinnon, Askanas, & Diamond, 2014; Morse, 1979; Nixon, Holmes, & Diamond, 2001), I determined the relative abundance of each tree species at Bicknell's Thrush presence sites and averaged the abundance across studies. Shrubs were removed because not all studies included them. Balsam fir (*Abies balsamea*) and paper birch (*Betula papyrifera*) had the highest relative abundance values of all tree species within all studies and showed average relative abundances above 20% across studies, indicating that these species are commonly co‐distributed with Bicknell's Thrush. Chisholm and Leonard (2008) also found that balsam fir and paper birch were commonly associated with Bicknell's Thrush, based on the mean of relative abundance and dominance (percentage of total basal area of that species, calculated by d.b.h. [diameter at breast height] measurements) for each tree species; raw abundance values were not reported for this study which prevented a direct abundance comparison.

Two of the published Gray‐cheeked Thrush studies (Kessel, 1998; Spindler & Kessel, 1980) contained importance values for tree species instead of relative abundance; the importance value for each species was calculated as the average of the relative frequency, abundance, and dominance of that species. Frequency represents the proportion of sites with that species, abundance is the proportion of individuals of that species, and dominance is the basal area of that species, measured from d.b.h. (see Curtis & McIntosh, 1951). Importance values for each tree species were calculated from FitzGerald, Whitaker, Ralston, Kichman, & Warkentin (2017), and then the importance value for each tree species was averaged across all three sources. Three species, balsam fir, black spruce, and white spruce had average importance values >20%, and were therefore included as species commonly associated with Gray‐cheeked Thrush.

I obtained tree species occurrence data through the Global Biodiversity Information Facility (www.gbif.org). The GBIF accrues species occurrence data from published records, museum collections, and citizen observations and makes these data freely available. Data were vetted and georeferenced as described for bird species occurrences. I compiled 320 occurrences for paper birch, 417 for balsam fir, 314 for black spruce, and 358 for white spruce (Appendices S1 and S3).

### 2.3 Abiotic and biotic environmental layers

Abiotic and biotic variables were included as environmental layers. In all, 19 modern bioclimatic layers based on climate data from 1950 to 2000 (Hijmans, Cameron, Parra, Jones, & Jarvis, 2005) were downloaded at a ~5 km^2^ resolution and clipped to cover North America to comprise the abiotic variables, hereafter referred to as “climate.” Four tree species (balsam fir, paper birch, black spruce, and white spruce) were tested as biotic correlatives to increase the predictability of my ecological niche models (Heikkinen et al., 2007; Wooten & Gibbs, 2012; de Araújo et al., 2014). Using occurrence data for tree species overlaid with the full set of 19 bioclimatic variables, I used maxent 3.3.3k (Phillips, Anderson, & Schapire, 2006) to produce probability distributions for each tree species (Appendix S2). maxent uses a maximum entropy algorithm to assess the niche conditions associated with presence‐only occurrences, and a habitat suitability model is created based on where those same conditions are found in geographical space (Elith et al., 2011; Phillips et al., 2006). The distribution model for white spruce resulted in a poor model (AUC = 0.827) and was not used in thrush ENMs. Habitat suitability scores for the three remaining tree species were used as environmental variables to model the ecological niches of the *Catharus* thrushes; hereafter, these distributional layers are called “trees.” Because tree distribution models rely heavily on the accuracy of the tree ENMs and the availability of occurrence data, I also tested the use of published tree range maps in niche modeling. Geographical range shapefiles were downloaded from http://gec.cr.usgs.gov/data/little (USGS, 1999), converted into ascii files based on presence (value of 1) and absence (value of 0), and then incorporated into the maxent model as categorical variables with the 19 bioclimatic variable set; hereafter, these layers are referred to as “shapefiles.” Tree habitat suitability scores and range shapefiles were not highly correlated (>0.7) with any bioclimatic variable. Because eliminating variables reduces the accuracy of the models (Elith et al., 2011; Sesink‐Clee et al., 2015), all variables were included in modeling.

### 2.4 Ecological niche modeling

Ecological niche models were run for a minimum of 25 replicates using maxent. For each replicate, 75% of the occurrence data was used to calibrate the model, and the remaining 25% was used to evaluate the model using the area under the curve of the receiver operating characteristic. The greater the area under the curve (the higher the AUC value), the better the model is at determining suitable versus unsuitable areas for the given data (Phillips et al., 2006). In general, AUC values below 0.7 are considered inaccurate and no better than random, whereas values above 0.9 indicate a high accuracy of the model to the data (Baldwin, 2009; Elith et al., 2011; Fielding & Bell, 1997; McFarland et al., 2013; Phillips et al., 2006). A jackknife test determined which variables contributed the most to each model. For each jackknife test, one variable was removed from the model and the results compared to the complete model; the removed variables that caused the highest drop in model performance were the variables that contributed the most to the model (Phillips et al., 2006).

I compared four types of models for both species to determine the best model to use for niche divergence tests: (1) climate only, (2) climate/trees, (3) climate/shapefiles, and (4) trees only. To compare model performance, I analyzed the AUC_test_ statistic using analyses of variance (ANOVAs) and Tukey's Honestly Significant Difference (HSD) post‐hoc test (de Araújo et al., 2014). Analyses were run in R (R Development Core Team, 2015) using the “MASS” package (Venables & Ripley, 2002).

### 2.5 Niche divergence tests

Actual niche overlap between Bicknell's Thrush and Gray‐cheeked Thrush was calculated using Schoener's *D* and the *I* statistic, sensu Warren et al. (2008), with the niche overlap tool in ENMtools; Schoener's *D* views the maxent output as species abundance values, whereas the *I* statistic assumes the output is a probability distribution. Overlap was calculated using ENMtools (Warren et al., 2008, 2010) by summing the differences between probability scores for each bird species at each pixel and subtracting those values from 1, after all probability scores have been standardized to a sum of 1. A value of 0 indicates no overlap, and a value of 1 means complete overlap.

To determine whether niche differences arise from niche divergence or simply from different background availability, I used the background similarity method in ENMtools (Warren et al., 2008, 2010). Because the background delineation can affect niche analyses (McCormack et al., 2009; Peterson et al., 2011; Warren et al., 2008), I compared three different background distributions, defined as the areas accessible to each species. First, I incorporated information on dispersal ability to create a buffer zone around known occurrence points (Barve et al., 2011; Peterson, 2011; Soberón & Peterson, 2005). Studds et al. (2012) examined hydrogen isotopic signatures from feathers of captured second‐year Bicknell's Thrush to compare first time breeding locales to natal area (where the feathers were grown). Most birds dispersed up to 200 km from their birthplace although ca. 4% of birds examined dispersed as much as 700 km (Studds et al., 2012). I therefore tested niche divergence for buffers of 200 and 700 km around occurrence points. I also used the minimum training presence threshold of maxent distribution models of each thrush species to define background distributions.

To estimate background niches, random points equal to the number of actual occurrence points were chosen from each background distribution. Background niche models were created in maxent from 75% of the random background points and calibrated with the remaining 25%, resulting in models showing the total accessible area for a species. Overlap between habitat suitability (actual) models created in maxent for one species and the background (accessible area) model of the other species was calculated, and vice versa (Figure 1); overlap was calculated for 100 replicates. These null background overlap values were then compared to actual overlap between the species. If actual overlap falls outside of the 95% confidence interval of the null background overlap values, the null hypothesis that differences in background niche availability account for niche differences is rejected. If actual overlap is much greater than the null background overlap, niche conservatism is supported. If actual overlap is much less than the null background overlap, niche divergence is supported (Warren et al., 2008, 2010).

For the multivariate analysis to test for niche divergence, background was defined using a 200 km buffer, 700 km buffer, or minimum training presence threshold from maxent outputs, as described above. Random background points (*n* = 1,000) were randomly chosen from each species’ background, and bioclimatic and tree distribution values from random background points and from actual occurrences were extracted using arcmap 10.3 (ESRI). A principal components analysis (PCA) with correlation matrix was conducted on actual and background occurrences for both species. The PCA reduced the variable set, and each principal component that explained at least 6% of the variance was analyzed, corresponding to PCs 1–3 and a total of 80% of the variance explained. The means of each PC for actual and background occurrences were calculated for each species, and the difference in means between background values (*d*_background_) and actual occurrence values (*d*_actual_) were compared for each PC (Figure 1). If species’ actual niche means are more divergent than expected based on background differences, niche divergence is supported along that niche axis. Niche divergence was therefore supported when the difference between the actual niche means was greater than the background niche difference, *d*_actual_ > *d*_background_ (Loera, Sosa, & Ickert‐Bond, 2012; McCormack et al., 2009). Multivariate tests were replicated 25 times with 75% of occurrences subsampled to assess significance. PCAs were performed in R and graphed using “ggplot2” (Wickham, 2009) and “vqv/ggbiplot” (Vu, 2011).

## 3 RESULTS

### 3.1 Evaluation of ecological niche models

Based on the ANOVA results and a visual comparison of models, the climate/trees model was considered the most accurate and was used for all further analyses of bird niche divergence (Figure 2). For Gray‐cheeked Thrush, the climate/trees model had significantly higher AUC scores than climate‐only, climate/shapefiles, and trees‐only models (Appendix S4). The climate/trees model also had the highest AUC score for Bicknell's Thrush although this score was not significantly higher than the climate‐only or climate‐shapefiles models. However, climate‐only and climate/shapefiles models tended to over‐project the potential distributions of the thrushes compared to models using climate/trees (Appendix S5). For example, the Bicknell's Thrush climate‐only and climate/shapefiles models showed some potential habitat in western New York, and the climate/shapefiles model also calculated expansive potential distribution in central Labrador and western Newfoundland, regions where they are not known to breed. For both thrushes, the models using trees‐only vastly over‐projected potential range; for example, the Gray‐cheeked Thrush trees‐only model projected the potential distribution into the northern Great Plains and the United States Appalachian Mountains.

### 3.2 Environmental differences and niche divergence tests

**Figure 3:**
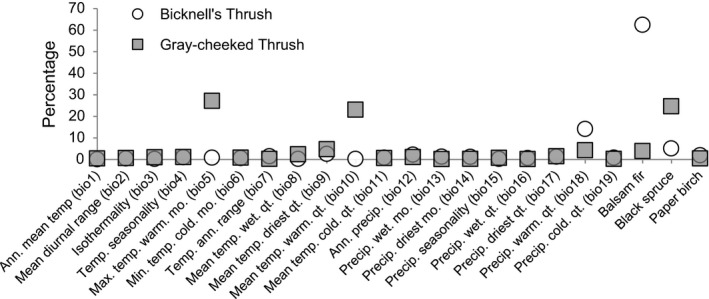
Percent contribution of each variable to the ecological niche model

Jackknife tests revealed that balsam fir and black spruce strongly influenced the respective niches of Bicknell's Thrush and Gray‐cheeked Thrush (Figure 3). Precipitation in the warmest quarter was the highest climatic contributor to the Bicknell's Thrush climate/trees model, whereas temperature in the warmest month and quarter contributed strongly for Gray‐cheeked Thrush.

Of 12 background similarity tests, eight (*p *<* *.05) supported niche divergence between Bicknell's Thrush and Gray‐cheeked Thrush; two additional tests supported niche divergence when *p *<* *.1 (Table 1). The two comparisons that did not reject niche conservatism (*p *>* *.1) compared Bicknell's Thrush occurrences to the Gray‐cheeked Thrush 200 km background, for both Schoener's *D* and the *I* statistic.

**Table 1:** Niche background test overlap values for the 200‐km buffer, 700‐km buffer, and minimum training presence backgrounds for Schoener's *D* and the *I* statistic. Niche conservatism was rejected when actual–actual overlap was significantly less than actual–background overlap

Backgrounds	Schoener's *D* (Actual overlap = 0.067)		*I* statistic (Actual overlap = 0.206)	
	*B*_actual_ to *G*_background_	*G*_actual_ to *B*_background_	*B*_actual_ to *G*_background_	*G*_actual_ to *B*_background_
200 km	0.062 ± 0.005	0.078 ± 0.004**	0.197 ± 0.013	0.219 ± 0.078*
700 km	0.088 ± 0.004***	0.171 ± 0.006***	0.273 ± 0.009***	0.370 ± 0.008**
Min.tr.pres.	0.083 ± 0.005***	0.073 ± 0.003**	0.254 ± 0.012***	0.221 ± 0.009*

***p <* *.01, **p <* *.05, *p <* *.1.

**Figure 4:**
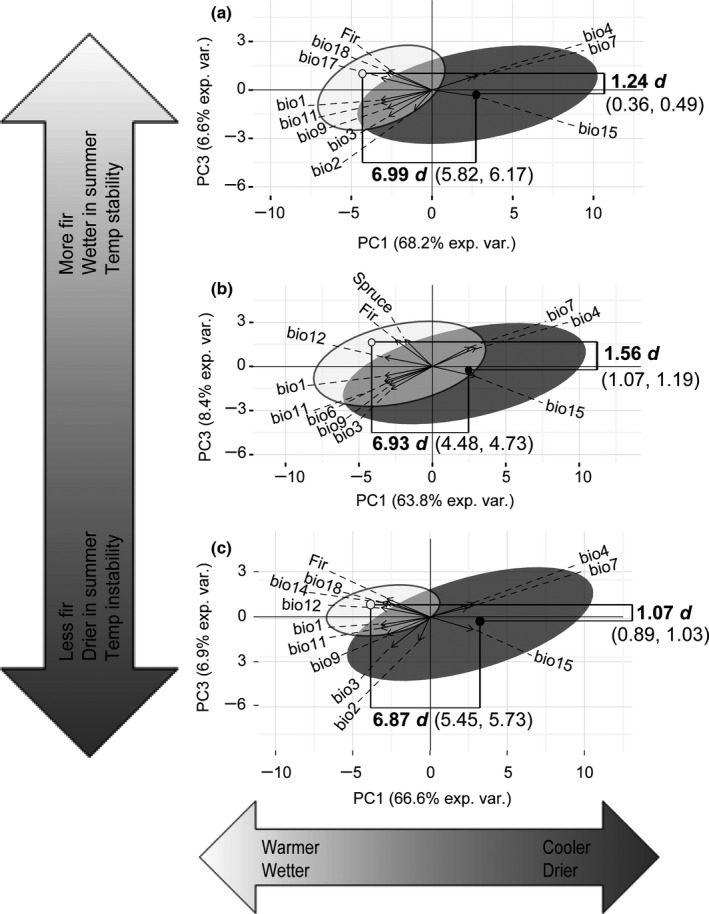
Multivariate niche tests for (a) 200 km, (b) 700 km, and (c) minimum training presence backgrounds. The backgrounds (95% probability ellipses around occurrence points), actual niche means (dots), and actual niche differences (significant values are bolded) for the divergent niche axes are shown for Bicknell's Thrush (white) and Gray‐cheeked Thrush (gray). The range of background differences, based on 25 replicates, is shown in parentheses. Niche divergence (indicated by d) was indicated when the actual niche difference was greater than the background difference. PC2 is not shown because actual niche differences were less than the background differences, supporting niche conservatism on that axis. The six variables with the highest loadings for each PC are shown. The bioclimatic full names may be found in Fig. 3

In the multivariate analysis, PC1, PC2, and PC3 explained >80% of the variance for all background conditions, with PC1 explaining 63.8%–68.2%. Significant niche differences were found for PC1 and PC3 for all background types (Figure 4). Analysis of the top variable loadings revealed that PC1 comprised annual temperature and precipitation variables, and showed that Bicknell's Thrushes occurred in warmer and wetter areas compared to Gray‐cheeked Thrushes. PC3 was defined by temperature stability, summer precipitation, balsam fir, and black spruce (700 km background only); Bicknell's Thrush often co‐occurred with balsam fir in wetter areas with more stable daily temperature ranges. PC2, defined by daily and annual temperature range and summer temperature, showed significant niche conservatism between the species for the 200 km background (actual difference = 0.44, background range = 0.91–1.12, variance explained = 11.28%) and the 700 km background (actual difference = 0.42, background range = 0.09–0.37, variance explained = 12.19%), but not for the minimum training presence threshold (actual difference = 0.66, background range = 0.49–0.72, variance explained = 12.71%).

## 4 DISCUSSION

### 4.1 Do the *Bicknell's Thrush* and *Gray‐cheeked Thrush* exhibit niche conservatism?

Ecological niche divergence occurs when populations adapt to different environments, whereas niche conservatism occurs when allopatric populations diverge in similar environments with no ecological selection. The Bicknell's Thrush and the Gray‐cheeked Thrush breed in superficially similar habitats, as would be expected if they diverged in allopatry in similar conditions and have retained ancestral niche characteristics. ENMs and multivariate analyses, however, reveal that the Bicknell's Thrush breeds in warmer, wetter, and more temperate locales with a high abundance of balsam fir, whereas Gray‐cheeked Thrush is found in drier, cooler areas, usually in the presence of black spruce. Although the Gray‐cheeked Thrush does occur in both spruce and fir in Newfoundland and Labrador (Thompson, Hogan, & Montevecchi, 1999; Whitaker, Taylor, & Warkentin, 2015; FitzGerald et al., 2017; see Appendix S2), balsam fir was not important for Gray‐cheeked Thrush across its entire range, indicating that the absence of balsam fir does not limit Gray‐cheeked Thrush distribution.

The two niche divergence tests employed here are fundamentally different in how they calculate niche divergence (niche overlap vs. niche means) and what they measure (species probability values from ENMs vs. environmental layer values at occurrence points). Nonetheless, I found evidence for niche divergence between the Bicknell's Thrush and Gray‐cheeked Thrush in 10 of 12 comparisons using the background similarity test and along 2 of 3 niche axes for each of three backgrounds using the multivariate niche analysis. Together, these comparisons validate that (1) the actual niches between the species are mostly distinct based on the environmental covariates utilized here and (2) these niche dissimilarities are not based solely on differences in background niche availability.

Niche divergence was not supported for two comparisons by the background similarity test and along niche axis 2 by the multivariate analyses. Backgrounds showed little geographic and niche overlap when the accessible area was defined by a 200 km buffer around occurrences, and the background similarity test showed that the Bicknell's Thrush actual niche was not significantly different than that of the Gray‐cheeked Thrush background niche (Table 1). A 200‐km buffer may have underestimated the background availability of these species; both species are long‐distance migrants, and Studds et al. (2012) determined that Bicknell's Thrushes occasionally disperse up to 700 km away from natal grounds. Furthermore, no verifiable occurrences for either species exist in a ~470 km gap along the north shore of the St. Lawrence and in eastern Quebec (Figure 2), but this may stem from poor surveying (e.g., Quebec Breeding Bird Atlas [http://www.atlas-oiseaux.qc.ca/], BBS [www.pwrc.usgs.gov/bbS/], eBird [www.ebird.org]). Niche models do not estimate a large gap, perhaps indicating that the species’ ranges may abut or overlap in this area. Obtaining occurrences (if they exist) from this gap and then re‐running the 200‐km background tests would show greater potential overlap between the species, which may then result in a rejection of the null hypothesis of niche conservatism. Although niche divergence was supported for all three backgrounds along PC1 and PC3, which together explained 72.2%–74.8% of the variance, niche conservatism was supported along PC2 for the 200‐ and 700‐km background tests. Because the Bicknell's Thrush and Gray‐cheeked Thrush are recently diverged sister species, some niche characteristics, such as summer temperature and daily and annual temperature range, may be ancestrally inherited.

Whether speciation results in niches that are divergent or conserved may be a simplistic representation of how populations diverge into species because a niche consists of multiple dimensions (Hutchinson, 1957), and selection can drive divergence along some dimensions, whereas ancestral characteristics may be conserved among others (Rundell & Price, 2009; Rundle & Nosil, 2005; Schluter, 2001). I found that most aspects of these species’ niches were divergent based on climate and tree species, but other aspects of their niche are unexplored, such as insect prey species consumed or plant species used as nesting material. For example, the thrushes may eat the same insect prey and their breeding distributions simply reflect that of their prey. Examining the stomach contents of breeding birds (i.e., actual prey species consumed) as well as the availability of prey (i.e., prey species available to be consumed) would show if eating habits differ between the species, and if they differ because of different food availability, but such data are hard to come by (e.g., Beal, 1915). The multivariate analyses showed that the thrush species were conserved along niche axis 2, defined as summer temperature and daily and annual temperature range. A temperature‐metabolism study of thrushes showed that Bicknell's Thrush had higher relative levels of oxygen consumption at lower temperatures, indicating that they are better adapted to colder temperatures than *Catharus* thrushes that breed in temperate climates (Holmes & Sawyer, 1973). Gray‐cheeked Thrush was not included in that study, but the Bicknell's Thrush and Gray‐cheeked Thrush show niche conservatism along a temperature axis (niche axis 2), perhaps indicating they may have similar physiologic constraints. This idea further emphasizes that some aspects of their niche may be ancestrally conserved while others are divergent.

Here, I examined niche divergence/conservatism on the breeding grounds of these Nearctic‐neotropical migrant species. However, divergent migration routes and wintering grounds have been shown to contribute to speciation and the maintenance of reproductive isolation in other species, including the Swainson's Thrush (*C. ustulatus*) (Delmore, Fox, & Irwin, 2012; Ruegg, 2007) and Eurasian Blackcap (*Sylvia atricapilla*) (Rolshausen, Segelbacher, Hobson, & Schaefer, 2009). The Bicknell's Thrush and Gray‐cheeked Thrush are long‐distance migrants that have different wintering grounds (Lowther et al., 2001; Rimmer et al., 2001), and speciation may have been promoted or maintained by adaptations to wintering grounds or migration allochrony (Winker, 2010). No study has examined whether these species exhibit niche conservatism or divergence on the wintering grounds or during migration.

### 4.2 Ecological selection played a role in speciation

Although niche conservatism implies speciation in allopatry, niche divergence tests alone cannot distinguish between (1) ecological speciation and (2) allopatric speciation followed by the accrual of niche differences (Warren et al., 2008). However, recently diverged species often have not had enough time to develop significant niche differences in the absence of strong ecological selection (Ahmadzadeh et al., 2013; Lovette & Hochachka, 2006; Peterson, 2011; Peterson et al., 1999). The Bicknell's Thrush/Gray‐cheeked Thrush and other co‐distributed boreal bird species complexes diverged during the Pleistocene, a geological era over the last ca. 2 my marked by repeated glaciation events that pushed boreal species into allopatric ice‐free refugia, promoting speciation (Weir & Schluter, 2004). If these thrushes did diverge in allopatry and niche differences accrued after speciation, other bird species complexes that diverged in Pleistocene refugia should show similar distributional patterns. However, the Bicknell's Thrush and Gray‐cheeked Thrush divide the boreal forest biome into northern and southeastern segments. Even if they are considered subspecies, there are still no other bird taxa that divide the boreal forest in a similar way. Because the Bicknell's Thrush is considered a full species (Monroe et al., 1995), it has the most restricted breeding range of any North American boreal bird species (Matteson, 2012). All other avifauna extend across the entirety of the boreal biome with genetic differentiation, if present, occurring in western North America, as shown in Fox Sparrows (*Passerella iliaca*) (Zink, 1996), Gray Jays (*Perisoreus canadensis*) (Dohms, 2016; van Els et al., 2012), Blackpoll Warblers (Ralston & Kirchman, 2012), and others (Weir & Schluter, 2004). Studies of Boreal Chickadees and Gray Jays found some population structure in Newfoundland, but they also found gene flow between populations (Dohms, 2016; van Els et al., 2012; Lait & Burg, 2013). The young age of the Bicknell's Thrush/Gray‐cheeked Thrush lineage (~410 kyr, Voelker et al., 2013) as well as the fact that no other boreal bird complex has a similar biogeographic break argues against a scenario in which allopatric speciation was followed by the accrual of niche differences (Peterson, 2011). A comprehensive historical biogeographic or phylogenomics study of the Bicknell's Thrush*/*Gray‐cheeked Thrush complex would determine whether these thrushes are reciprocally monophyletic, if any gene flow occurs, and whether selection has caused genomic divergence.

### 4.3 Use of biotic factors in ecological niche modeling

Biotic factors are often important in defining a species’ realized niche (de Araújo et al., 2014; Heikkinen et al., 2007; Pearson & Dawson, 2003), and here I included three tree species that frequently co‐occur with Bicknell's Thrush or Gray‐cheeked Thrush. The tree species layers were not significantly correlated with any climatic variable, indicating that these biotic variables are introducing new material into the models, a result also found by de Araújo et al. (2014). Models that included both abiotic (climate) and biotic (trees) variables had the highest AUC values and showed a tighter geographic overlap to the occurrence points compared to the climate‐only and climate/shapefiles models although differences between these three models were small. The real utility of using both climate and trees is revealed by the explanatory power of the tree layers (Figure 3). maxent calculates how varying the value of each environmental variable affects the probability of detecting the target species, revealing that Bicknell's Thrush is most likely to be present in areas where the abundance value of balsam fir is >0.44, corroborating with habitat studies surveyed for this project (Appendix S2). maxent shows that Gray‐cheeked Thrush occurs in areas where black spruce is present (abundance value > 0.1), and habitat studies show that black spruce has a high importance value for Gray‐cheeked Thrush. However, the biotic‐only (trees) models performed poorly, showing that thrushes are not only responding to the tree species included in this study. In conclusion, ecological niche models that include biotic variables result in tighter distribution models and better define the relevant ecological variables that affect species’ distributions.

Species range maps are often readily available as downloadable shapefiles and simpler to add to ENMs than creating probability distribution layers based on occurrence data. However, range maps assume a uniform abundance of that species across the range (i.e., presence vs. absence), whereas probability distributions are more likely to mirror actual conditions. In the Bicknell's Thrush climate/shapefile model, balsam fir was the variable that had the highest contribution although this value (32.5%) is much less than the climate/trees model (62.5%); tree shapefiles were not important contributors for the Gray‐cheeked Thrush model despite the fact that black spruce was a strong contributor for the combined model and had a high importance value. Furthermore, climate/shapefiles models had lower AUC values and over‐projected distributions compared to climate/trees models. Range shapefiles should be used only when occurrence data are not available.

## 5 CONCLUSION

The recently diverged Bicknell's Thrush and Gray‐cheeked Thrush breed in boreal forests in superficially similar habitats, and I expected to show that these thrushes exhibit niche conservatism, as expected if they diverged in allopatry and ecological divergence did not play a large role in speciation. Niche divergence tests, however, show that Bicknell's Thrush and Gray‐cheeked Thrush inhabit significantly different breeding niches and these niche differences are not based solely on niche availability. Although these tests cannot directly determine whether adaptive ecological divergence caused speciation, the Bicknell's Thrush and Gray‐cheeked Thrush have a unique distribution among boreal birds and exhibit significant niche divergence, indicating that ecological divergence may have played a significant role in their speciation. Future genome‐scale studies may reveal the specific loci that have been the target of ecological selection. Finally, this study validates the inclusion of relevant biotic factors in ecological niche modeling to increase model accuracy.

## CONFLICT OF INTEREST

None declared.

## DATA ACCESSIBIILITY

Occurrence points are freely accessible (see Appendix S1), and the WorldClim bioclimatic layers are available through www.worldclim.org. All habitat suitability models generated in this study will be available as raster grids from the Pangaea database, and will be uploaded upon acceptance of this manuscript.
