# Supplementary material for: Division within the North American boreal forest: Ecological niche divergence between the Bicknell's Thrush (Catharus bicknelli) and Gray‐cheeked Thrush (C. minimus)
Source: Ecol Evol. 2017 Jun 8;7(14):5285–95. doi: 10.1002/ece3.3080 (PMC5528206; doi:10.1002/ece3.3080)

FitzGerald, A.M. (2017) Division within the North American boreal forest: ecological niche divergence between the Bicknell's Thrush (*Catharus bicknelli*) and Gray-cheeked Thrush (*C. minimus*). Ecology and Evolution.

Supporting Information

APPENDIX S1. Sources and record types of thrush and tree occurrences. Source names are listed at the end of the table.

	Type of record	n	Source*	Catalog no./Atlas sq./Survey rte.
Bicknell's Thrush (n = 274)	Audiovisual	27	BLB	5898, 17542, 17543
			CUML	4211, 77096, 100908, 102272, 106863, 136157, 140078, 195518
			eBird	S14750598, S18967683, S24066689, S24191960, S11226526, S18838552, S18949800, S18958179, S19110775, S22709234
			FLMNH	13803, 18360, 18366, 18463, 18481
			XC	22196
	Preserved specimen	41	AMNH	DOT-13282, DOT-13293, DOT-13305, DOT-13310, DOT-13320, DOT-13328, DOT-13328, DOT-13383
			CMN	62216, 62219
			NYSM	zo-10982, zo-11138, zo-11235, zo-11251, zo-13331, zo-14314, zo-15341, zo-4003, zo-4004, zt-1004, zt-1032, zt-1106, zt-1109, zt-1402, zt-1407, zt-1412, zt-1417, zt-1419, zt-1433, zt-1436, zt-1441, zt-1451, zt-1460, zt-1486, zt-1490, zt-1500, zt-561, zt-956, zt-988, zt-993
			YPM	ORN 098825
	Literature	1	Lit. 1	Marshall 2001
	Survey	193	BBA-NY	5382A, 5383B, 5384C, 5467B, 5483A, 5485A, 5487D, 5589B, 5589D, 5590D, 5593B, 5667B, 5668D, 5688C, 5688D, 5689C, 5783A, 5787B, 5789D, 5790A, 5889B, 5891A, 5894A, 5895C, 5895D, 5986A, 5986C, 5987A, 5988A, 5988B, 5990D, 5995B, 5265D, 5365B, 5464A, 5464C, 5465A, 5465B, 5465D, 5566B, 5567D, 5666C, 5666D, 5766A
			BBS	124-76-39, 124-76-39, 124-76-39, 124-76-39
			BBA-ME	163, 199
			BBA-QC	19CN53, 19CN55, 19CP31, 19CP68, 19CP92
			MBA	19EN69, 19EN89, 19EN89, 19EP80, 19EP90, 19EP91, 19FN64, 19FN64, 19FN64, 19FN71, 19FN72, 19FN72, 19FN72, 19FN72, 19FN72, 19FN72, 19FN73, 19FN83, 19FN83, 19FN83, 19FN83, 19FN84, 19FN84, 20LR23, 20PS66, 20PS67, 20PS67, 20PS67, 20PS67, 20PS67, 20PS67, 20PS74, 20PS74, 20PS74, 20PS75, 20PS76, 20PS86, 20PS87, 20PS87, 20PS87, 20PT90, 20PT90, 20PT90, 20PT90
			MBW^	S3043798, S3043841, S3043996, S3043919, S3043916, S3043879, S3043960, S3043910, S3044054, S3043985, S3753174, S3044091, S3043836, S3043923, S3043833, S3043773, S3043864, S3043956, S3043964, S5135493, S3043795, S3043952, S3043926, S3043815, S3043979, S3053517, S3043803, S3043995, S3043777, S3043861, S11112496, S3043794, S3043970, S3043983, S3043966, S3043940, S3044090, S3043758, S23634564, S3753185, S3044040, S3043860, S3043934, S3043948, S3043810, S3043989, S3043939, S3043932, S3043930, S3043874, S3043847, S3043761, S3043780, S18969282, S3043998, S3043978, S3043747, S3043837, S3043971, S3044085, S3043751, S3044001, S3043887, S3043866, S3043856, S5118214, S3043942, S3043749, S3043884, S3043891, S3043850, S3043783, S3043929, S3044003, S3043957, S3044009, S3043793, S3043835, S3043767, S3044010, S3043896, S3043753, S3043757, S3043969, S6584609, S3043933, S3043772, S3044039, S3043905
			PNMT	eBird S20510694, eBird S20510476, eBird S20510642
			SONA	eBird S17367033
	Observation	12	eBird	S11044400, S1178115, S1219762, S1219975, S1219981, S14560176, S17218444, S18842925, S18885569, S19103232, S6519621, S8577535

Gray-cheeked Thrush (n= 534)	Audiovisual	24	BLB	BLB21281
			CUML	4206, 49579, 67350, 105800, 132168, 176383, 176393, 193472, 193520, 462555, 463051, 464037
			eBird	S14543285, S24280843, S5210079
			FLMNH	4571, 18124, 18178, 18187, 18537, 18552, 18553, 18554
	Preserved specimen	114	AMNH	DOT-13329, DOT-13332
			CM	P136329, P136405, P137756
			CMN	35972, 43702, 43705, 43707, 43710, 59003, 61557, 68123, 68124, 77727, 77730, 79579, 79581, 80179, 80181, 80188, 80189, 80191, 80193, S1453, S1467, S1713
			CRCM	56-151, 56-204, 56-220
			MSB	24804
			MVZ	156004, 158368, 158370
			NYSM	YG_1, zt-1170, zt-1184, zt-1195, zt-1198, zt-1208, zt-1210, zt-1221, zt-1232, zt-1236, zt-1239, zt-1240, zt-1242, zt-1250, zt-1252, zt-1253, zt-1264, zt-1265, zt-1270, zt-1272, zt-780, zt-782, zt-788, zt-797, zt-797, zt-952
			OSUM	Birds E 2098
			PMAE	349397, 349401
			RBCM	16839, 16840, 16841
			ROM	94523, 95111, 110698, 116071, 116157, 116309, 124184, 125061
			SBMNH	30950
			SNOMNH	15551
			UAM	651, 657, 1036, 2234, 4443, 4452, 4454, 5032, 5930, 7412, 7440, 7457, 7475, 13406, 13410, 14546, 18687, 19812, 21198, 21947, 27375, 31509
			UWBM	40300, 82277
			WFVZ	61831, 85685, 227802
			YPM	ORN 141002, ORN 141003, ORN 141005, ORN 141547, ORN 141548, ORN 141573, ORN 141579, ORN 141582, ORN 141583, ORN 141585, ORN 141598
	Literature	1	Lit.2	
	Survey	369	BBS	124-57-15, 124-57-36, 124-57-36, 124-57-36, 124-57-4, 124-57-4, 124-57-9, 124-04-225, 124-04-225, 24-04-225, 124-04-225, 124-04-225, 124-04-225, 124-04-225, 124-11-137, 124-11-137, 124-11-137, 124-11-339, 124-11-339, 124-11-339, 124-11-339, 124-43-12, 124-43-12, 124-43-12, 124-43-3, 124-43-3, 124-43-3, 124-43-3, 124-43-3, 124-56-100, 124-56-100, 124-93-164, 124-93-164, 124-93-164, 124-93-164, 24-93-164, 124-93-164, 124-93-164, 124-93-164, 124-93-164, 124-93-19, 124-93-19, 124-93-19, 124-93-19, 124-93-26, 124-93-26, 124-93-26, 124-93-26, 124-93-27, 124-93-29, 124-93-29, 124-93-29, 124-93-31, 124-93-314, 124-93-314, 124-93-36, 124-93-43, 124-93-43, 124-93-51, 124-93-55, 124-93-55, 124-93-6, 124-93-64, 124-93-64, 124-93-64, 124-93-66, 124-93-66, 124-93-66, 124-93-66, 124-93-66, 124-93-66, 124-93-66, 124-93-78, 124-93-78, 124-93-78, 124-93-78, 124-93-78, 124-93-78, 124-93-78, 124-93-79, 124-93-79, 124-93-79, 840-03-1, 840-03-1, 840-03-1, 840-03-1, 840-03-1, 840-03-118, 840-03-231, 840-03-231, 840-03-231, 840-03-30, 840-03-318, 840-03-318, 840-03-4, 840-03-4, 840-03-409, 840-03-420, 840-03-420, 840-03-420, 840-03-51, 840-03-51, 840-03-53, 840-03-53, 840-03-53, 840-03-60, 840-03-60, 840-03-60, 840-03-60, 840-03-7, 840-03-7, 840-03-7, 840-03-7, 840-03-7, 840-03-7, 840-03-8, 840-03-8, 840-03-8, 840-03-8, 840-03-8, 840-03-883, 840-03-902, 840-03-902
			LGL	(eBird) S19364800, S19364815, S19364834, S19364853, S19364921, S19364951, S19365098, S19365130
			NTNU	93, 94, 2195, 2206, 2328, 2329, 2333, 2335, 2338, 2339, 2342, 2343, 2344, 2347, 2381, 2388, 2389, 3375, 3660, 3706, 3728, 3749, 3770, 3771, 3772, 3778, 3810, 3816, 4145, 4221, 4346, 5589, 6414, 6426, 6429, 7015, 7016, 8872, 9118, 9183, 9247, 9249, 9265, 9266, 9395, 9508, 9720, 9725, 11073, 12630, 12635, 12638, 12639, 12641,12643, Beaverlodge Lake, Great Bear Lake SE

			BBA_BC	08MM12, 309721, 309886, 309895, 310793, 310795, 311444, 311598, 311648, 313052, 313064, 313068, 313236, 313244, 321852, 321948, 324013, 325095, 325160, 325174, 325183, 325196, 326186, 326190, 326196, 327273, 329453, 329455, 329466, 329471, 329549, 337643, 337826, 338186, 338229, 338330, 338387, 338404, 338408, 339731, 339876, 339892, 339893
			BBA_MB	530191, 505058, 508245, 508247, 508438, 508444, 508479, 508489, 508510, 509097, 509111, 509137, 511394, 512463, 512581, 512583, 512594, 512597, 512600, 512602, 512615, 512656, 512661, 512668, 512696, 512813, 512820, 513019, 513063, 513078, 513096, 513115, 513129, 513196, 513317, 513361, 513365, 513373, 513377, 513385, 513446, 513465, 513471, 513482, 513488, 513521, 513522, 513524, 513525, 513799, 513848, 514376, 514476, 514598, 515331, 515915, 516324, 516325, 516331, 516562, 516572, 521158, 521326, 525647, 525708, 525722, 525787, 525799, 525868, 526047, 526062, 526345, 526464, 526545, 526561, 526705, 526711, 526757, 527471, 527491, 527492, 527513, 528277, 528320, 528341, 528505, 528522, 528527, 528546, 528556, 528565, 528580, 528610, 528715, 528744, 529920, 530013, 530112, 530164, 530172, 530184, 530208, 530234, 534811, 534885, 534886, 534900, 535101, 535106, 535539, 535696, 535702, 536645, 536655, 536905, 536909, 536933, 536955, 536961, 536982, 537030, 537039, 537041, 537048, 537054, 537726, 537732, 537884, 537920, 537942, 540773, 540780, 540786
			BBA_ON	1462, 58990
			BBA_QC	19EE33, 19EE34, 19EE35
	Observation	26	eBird	S10777976, S11198370, S11217354, S11710836, S14490163, S14883898, S17175624, S23907842, S24316986, S2590547, S2654125, S2664026, S2665971, S2671008, S2698113, S2698152, S2864207, S2865208, S2879631, S2902039, S34374833, S4388468, S4979667, S7275352, S8448340, S8600375
Balsam fir (n = 417)	Audiovisual	0		
	Preserved specimen	332	ACAD	11710, 16941, 19548, 27393, 27395, 27396, 27399, 32630, 32920, 34313, 34314, 34315, 34316, 34317, 37108, 39839, 40529, 40530, 40531, 40532, 40533, 40534, 40535, 40536, 40537, 40538, 40539, 40540, 40541, 40542, 40543, 40544, 40545, 40546, 40548, 40549, 40550, 40551, 40552, 40553, 40554, 40556, 40557, 40558, 40559, 40560, 40561, 40562, 40564, 43203, 43452, 43453, 43454, 44904, 46498, 46499, 46500, 49071, 49763, 49919, 52168, 60313, 60906, 63017, 64239, 65902, 67539, 67720, 69742, CBU1089, CBU1089A, CBU1696, CBU229, CBU230, CBU231, CBU232, CBU234, CBU235, CBU2539, CBU2540, CBU3301, CBU3385, CBU3600, CBU3602, CBU3864, ECS012366, ECS012958, ECS015145, ECS021624, ECS021625, ECS021628
			CMN	CAN 5742, CAN 584186
			CONN	CONN00012062, CONN00072601, CONN00074307, CONN00085644, CONN00135821, CONN00136119, CONN00136845, CONN00142764, CONN00149288, CONN00169376, CONN00175008, CONN00175009, CONN00175010, CONN00175011, CONN00175012, CONN00175013, CONN00175014, CONN00175016, CONN00175017, CONN00175020, CONN00175021, CONN00175022, CONN00175023, CONN00175024, CONN00175026, CONN00175029, CONN00175030, CONN00175031, CONN00175032, CONN00175033
			KU	153089, 350142, 353769, 354435
			MO	699608, 700007, 1277892
			NYBG	1087471
			RBGE	19762145, 19762146, 19870551, 20070959, 20090340
			ROM	31653, 31656, 31661, 52406, 87613, 96574, 108133, 111109, 127973, 138292, 229099, 231238, 254314, 254425, 254468
			SNSB	GLM-F036294 / 102256 / 64971
			UABM	449, 450, 451, 453, 455, 456, 460, 461, 462, 469, 470, 25540, 26168, 27916, 28060, 29133, 33220, 33222, 33232, 33234, 39475, 49295, 56471, 57386, 59474, 81829, 84490, 94325, 99844, 101680, 104987,112841, 114052, 124860, 124861, 124862, 124863, 124864, 124865, 124866, 124867, 124868, 124869, 124870, 124871, 124872
			UNA	UNA00060801
			UBC	V174554, V205845, V218905

			UL	QFA0081903, QFA0131437, QFA0131490, QFA0160355, QFA0167107, QFA0182356, QFA0184924, QFA0188872, QFA0230860, QFA0249027, QFA0264321, QFA0282398, QFA0312941, QFA0346285, QFA0357461, QFA0357463, QFA0357464, QFA0357468, QFA0357469, QFA0357472, QFA0357473, QFA0384255, QFA0387161, QFA0397800, QFA0399587, QFA0404978, QFA0404979, QFA0404980, QFA0404981, QFA0404982, QFA0404983, QFA0404984, QFA0404985, QFA0410889, QFA0428968, QFA0429230, QFA0450989, QFA0455661, QFA0467160, QFA0468728, QFA0477778, QFA0477835, QFA0482369, QFA0482370, QFA0482501, QFA0490985, QFA0492141, QFA0495445, QFA0496346, QFA0498564, QFA0501274, QFA0509247, QFA0533574, QFA0534079, QFA0545229, QFA0545578, QFA0545581, QFA0545582, QFA0545583, QFA0545584, QFA0545585, QFA0545783, QFA0545784, QFA0547214, QFA0558099, QFA0558107, QFA0558108, QFA0570841, QFA0577983, QFA0578027, QFA0578055, QFA0578070, QFA0578102, QFA0578155, QFA0579342, QFA0586945, QFA0586946, QFA0588101, QFA0594049, QFA0596497, QFA0596670
			UMB	20022, 20023, 20024, 20025, 20026, 20027, 20028, 23729, 23751, 27895, 34067, 34087, 43583, 45031, 45401, 51282, 51607, 51736, 53570, 53842, 53843, 54934, 55327, 56761, 60216
			UMBC	MT00000857, MT00000926, MT00001026, MT00001193, MT00002214, MT00012992, MT00018165, MT00023190, MT00045594, MT00066437, MT00097112, MT00109176, MT00123186, MT00123332, MT00123582, MT00127779, MT00128071, MT00128672, MT00179569, MT00180219, MT00180272
			UPS	V-148168
			USU	39854
			YPM	CBS 029238
	Literature	0		
	Survey	37	Lit_3	Blackdome2, Blackdome5, Cascade1, Cascade2, Cascade4, Coburn3, Coburn4, Coburn6, Cook'sHarbour1, Esker2, Madawaska1, Madawaska4, Mansfield2, MoneyPoint1, MoneyPoint2, Reddington2, RedRocks2, S.S. Bog8, Sugarloaf2, Sugarloaf4
			Lit_4	CandlestickPond1, CandlestickPond3, CandlestickPond5, LaManche1, LaManche3, LaManche5, MainRiver1, MainRiver3, MainRiverRoost1, MainRiverRoost4, MainRiverRoost6, PatricksCove1, PatricksCove2, PatricksCove6, Starlight2, Starlight3, Starlight5
	Observation	48	iNat	71928, 127754, 133295, 151858, 178698, 182120, 187377, 187932, 191117, 196242, 197005, 197837, 207902, 208033, 214022, 214207, 217199, 222135, 254793, 265205, 272433, 274155, 285258, 307747, 309535, 316412, 335965, 338372, 347253, 347617, 347944, 349673, 358936, 370145, 373887, 391835, 394273, 398767, 403179, 405259, 426384, 426387, 427711, 427715, 457638, 477788, 489560, 489561
Black spruce (n = 314)	Audiovisual	0		
	Preserved specimen	284	ACAD	19645, 20222, 23590, 27419, 27424, 30841, 40630, 40639, 40647, 60018, 67351, 68983, 71607, CBU246, ECS008062, ECS015692, ECS021640
			CMN	CAN 202490, CAN 216121, CAN 258551, CAN 260885, CAN 260886, CAN 262134, CAN 263810, CAN 268239, CAN 275880, CAN 279112, CAN 297506, CAN 310721, CAN 310723, CAN 310724, CAN 329574, CAN 399240, CAN 407588, CAN 445711, CAN 568758, CAN 568759, CAN 568760, CAN 568761, CAN 568762, CAN 568766, CAN 568771, CAN 568783, CAN 6323, CAN 6324, CAN 6325, CAN 6326, CAN 6327, CAN 6328, CAN 6329, CAN 6330, CAN 6331, CAN 6332, CAN 6333
			FM	27043
			FMNH	1557870
			KU	159395, 350094
			LSU	71405, 71406
			MO	700020, 1682889, 2881607, 2906780
			NMNH	3466605.102, 3524073.102, 3616569.102, 3616573.102, 3616575.102
			NPGS	1654186
			ROM	16109, 53417, 87622, 110784, 111105, 129132, 129133, 220655, 221443, 221701, 221716, 254212, 254312

			UABM	579, 580, 581, 582, 583, 584, 586, 21904, 22631, 25299, 26476, 28030, 28160, 29204, 30549, 32813, 33199, 38905, 40041, 40693, 45411, 55659, 56245, 71596, 78060, 78908, 78968, 81294, 81761, 81802, 81828, 82095, 82105, 82108, 90658, 94329, 98901, 99913, 102471, 102472, 104990, 104991, 106178, 112843, 112844, 114075, 114620, 114872, 119433, 121334, 121725, 123021, 123021, 125925, V12-186-20
			UBC	V109669, V163889, V196273, V216919
			UNA	UNA00063657
			UAM	13402, 15873, 26479, 26480, 26481, 33525, 35542, 51546, 55751, 56460, 57385, 57802, 57828, 62143, 62331, 62505, 64054, 64329, 64676, 65407, 65548, 65673, 65682, 65781, 67487, 67633, 67928, 69008, 69371, 69392, 70922, 72750, 72802, 73094, 76570, 77580, 81082, 87264, 87463, 87656, 90567, 90594, 91205, 91303, 91875, 114544, 131250, 131897, 138958, 141397, 144437, 196497, 248549
			CONN	CONN00012196, CONN00012211, CONN00012224, CONN00012226, CONN00012227, CONN00012231, CONN00072291, CONN00085831, CONN00136244, CONN00137443, CONN00142050, CONN00153444, CONN00173917, CONN00173927, CONN00173931
			UL	QFA0066673, QFA0182351, QFA0186058, QFA0230956, QFA0264279, QFA0264303, QFA0278437, QFA0278438, QFA0312955, QFA0312956, QFA0412267, QFA0412271, QFA0412272, QFA0438900, QFA0452053, QFA0458894, QFA0478198, QFA0482367,QFA0490994, QFA0496348, QFA0498565, QFA0509460, QFA0509504, QFA0546752, QFA0546753, QFA0546756, QFA0570421, QFA0596320, QFA0596662
			UMB	19956, 19957, 19958, 19959, 19960, 19961, 19963, 19964, 19965, 19966, 23727, 23763, 23769, 24331, 25336, 26740, 29061, 30881, 38595, 43431, 45145, 45367, 51603, 53822, 54304, 57323, 62920, 69667, 71157, 71691
			UMBC	MT00022113, MT00023191, MT00024238, MT00025757, MT00109179, MT00123188, MT00123244, MT00123254, MT00123342, MT00127990, MT00128072, MT00179564, MT00180250, MT00180256
	Literature	0		
	Survey	18	Lit_3	Bog4, Cook'sHarbour1, Esker2, MainRiver1, MainRiver3, S.S. Bog8
			Lit_4	CandlestickPond4, LaManche1, LaManche3, LaManche5, MainRiverRoost1, MainRiverRoost4, MainRiverRoost6, Orma1, Orma3, Patrick'sCove2, Starlight3, TwinFalls1
	Observation	12	AD	4294809, 4294810, 4295086, 4295087, 4295089, 4295090, 4295091, 4295092, 4295095
			iNat	74925, 182069, 398538
White spruce (n = 358)	Audiovisual	1	iNat	349672
	Preserved specimen	356	ACAD	10113, 19643, 20666, 20826, 22569, 27407, 31559, 32629, 34299, 34300, 40590, 40591, 40594, 40603, 40606, 40611, 41553, 43543, 49759, 60768, 62980, 64264, 67661, 71699, 71873, CBU1096, CBU1698A, CBU239, CBU3483, ECS021638
			CMN	CAN123329, CAN127428, CAN151872, CAN200053, CAN211352, CAN216120, CAN225999, CAN260884, CAN263809, CAN268231, CAN268232, CAN271608, CAN275876, CAN279824, CAN281095, CAN297507, CAN310722, CAN329572, CAN329573, CAN329576, CAN407593, CAN457169, CAN568670, CAN568684, CAN568685, CAN568687, CAN568705, CAN582913, CAN584219, CAN586794, CAN586919, CAN6202, CAN6219, CAN6221, CAN6222, CAN6224, CAN6227, CAN6228, CAN6245, CAN6246, CAN6250, CAN6251, CAN6252, CAN6260
			EMBL	EU701140, JN999372
			FM	27017, 27018, 27020, 33223
			FMNH	1557866
			KU	117741, 117742, 156899, 156906, 156907, 156908, 340479, 354155, 355370
			LSU	71400
			MO	699647

			NMNH	3525533, 3616517, 3616518, 3616520, 3616521, 3616522, 3616523, 3616524, 3616525, 3616528
			NYBG	30217, 30220, 30221, 30222, 30239, 30240, 30241, 30255, 30265, 30266, 30267
			RBGE	20070946
			RBGK	K000340475, K000340478, K000340481, K000340482, K000340484, K000340485, K000340486, K000340488, K000340489, K000340491, K000340511, K000340514, K000554026
			ROM	31687, 31693, 88781, 110785, 222307, 229044, 231284, 254313, 254426, 260907
			SNSB	M-0140484 / 574678 / 156637
			UABM	566, 567, 568, 569, 570, 571, 572, 573, 578, 22665, 22666, 26269, 26344, 26462, 28634, 29136, 29196, 33202, 33203, 39459, 40318, 41234, 45860, 52861, 53718, 55658, 71858, 71863, 78734, 78736, 78737, 78741, 81295, 81827, 82111, 83587, 88277, 88384, 98183, 99015, 99234, 100339, 100866, 104321, 104988, 104989, 108038, 108038, 110418, 112842, 114727, 115273, 119685, 121721, 121723, 121724, 126923
			UAM	2941, 6580, 15939, 26477, 26478, 48286, 51585, 54022, 57226, 57249, 57881, 57897, 58144, 60883, 61631, 61651, 62211, 62329, 62450, 62593, 64458, 64610, 65456, 65624, 65832, 66264, 66333, 66466, 66866, 67488, 67514, 68352, 68869, 68881, 69384, 70024, 70607, 70965, 72206, 72421, 72737, 75385, 76426, 76760, 77407, 78577, 86316, 86369, 87227, 87240, 88075, 88436, 90925, 91302, 91398, 91619, 130931, 131173, 133358, 133788, 135473, 137991, 139355, 147851, 201041, 201042, 201073
			UAZ	dbsn295984
			UBC	V100006, V100052, V100053, V112402, V136961, V136962, V173982, V179400, V196272, V198139, V216921, V217928, V218892, V3261
			CONN	CONN00012160, CONN00012162, CONN00013239, CONN00130745
			UL	QFA0100878, QFA0102067, QFA0131583, QFA0131590, QFA0264323, QFA0328296, QFA0357628, QFA0357633, QFA0399582, QFA0410892, QFA0412255, QFA0412256, QFA0412263, QFA0429492, QFA0450987, QFA0466678, QFA0467161, QFA0478204, QFA0482372, QFA0490987, QFA0495447, QFA0496347, QFA0498556, QFA0502732, QFA0502740, QFA0509231, QFA0509304, QFA0512925, QFA0534083, QFA0547215, QFA0579569, QFA0579570
			UMB	19951, 19952, 19953, 19954, 19955, 23700, 23759, 23760, 29058, 42304, 43432, 47017, 47511, 48159, 53820, 53823, 54076, 54077, 57317, 63548, 69663, 70356, 71514, 71521, 73765
			UMBC	MT00001526, MT00006343, MT00012456, MT00024207, MT00024379, MT00123255, MT00123416, MT00127685, MT00128026, MT00128334, MT00179591, MT00180218, MT00180335, MT00187464
			NPGS	1654183
			UWBM	38115, 38132, 38136
	Literature	0		
	Survey	0		
	Observation	1	NG	-1969767624
Paper birch (n = 320)	Audiovisual	1	VU	SB569
	Preserved specimen	296	ACAD	20627, 24676, 24904, 26406, 26407, 26408, 28581, 28631, 30377, 30379, 35527, 39291, 39866, 42230, 42789, 42795, 42796, 43133, 47064, 47849, 49154, 51186, 51439, 52113, 54499, 55105, 59614, 60150, 64286, 69325, 69343, 72334, ECS004295, ECS007543, ECS008023, ECS019045, ECS029117, ECS029118, ECS029122, ECS029123, ECS029126, ECS029127
			CMN	CAN267581, CAN270477, CAN279731, CAN407601, CAN415108, CAN41918, CAN42042, CAN42053, CAN435841, CAN436201, CAN482910, CAN50970, CAN540651, CAN540861, CAN572537, CAN572595, CAN572604, CAN584369, CAN584370, CAN584371, CAN584417

CONN	CONN00017527, CONN00017531, CONN00017532, CONN00017534, CONN00017656, CONN00017666, CONN00017667, CONN00017676, CONN00017677, CONN00017685, CONN00017690, CONN00017693, CONN00017698, CONN00017705, CONN00017706, CONN00017951, CONN00022947, CONN00045917, CONN00045918, CONN00051130, CONN00051131, CONN00054462, CONN00059885, CONN00077130, CONN00098289, CONN00136276, CONN00139475, CONN00142616, CONN00152125
CSU	208106
EMBL	EU701116, EU701117, EU701118
ESU	18797
FMNH	152563
KU	203170, 203171, 203172, 203173, 203174, 203175, 203176, 203177, 203178, 203179, 203180, 203182, 203191, 203201, 203202, 203204, 203205, 203207, 354948, 355371, 368313
LEA	1337, 19271, 19361, 1301
MO	700739, 700767
MSU	299283
NMNH	3666235, 10240442
NYBG	42121
ORE	ORE25506, OSC99047
UL	QFA0002959, QFA0112688, QFA0167113, QFA0222406, QFA0250921, QFA0250944, QFA0264319, QFA0264359, QFA0288011, QFA0301033, QFA0314194, QFA0314196, QFA0364360, QFA0364367, QFA0364375, QFA0364391, QFA0364395, QFA0406076, QFA0419433, QFA0451096, QFA0456955, QFA0459018, QFA0459779, QFA0466525, QFA0466526, QFA0477818, QFA0482338, QFA0495190, QFA0503217, QFA0513574, QFA0533773, QFA0545627, QFA0545636, QFA0577510, QFA0590668, QFA0590671, QFA0590677, QFA0590678, QFA0590680
RBGE	19961304, 20090347
ROM	33159, 70219, 74458, 81460, 82930, 84692, 89648, 112383, 119980, 138287
UABM	78352, 114705, 115919, 121096, 121430, 125151, 125152, 125152, 126962, 134724
UAM	4689, 15880, 22726, 22728, 50817, 54317, 63351, 63401, 64478, 67708, 67716, 73370, 75298, 77486, 81395, 109562, 109590, 109630, 133309, 141496, 141496, 147835, 212225, 212257
UBC	V101453, V103749, V105955, V105963, V111464, V111467, V117602, V122123, V130958, V131135, V131136, V133687, V133898, V136539, V173758, V174777, V176682, V181181, V195637, V212867, V227832, V37791, V60812, V61420, V6487, V69622, V72235, V87969, V88060, V89680
UMB	6385, 6527, 23703, 23704, 24826, 25334, 25340, 35060, 45030, 47859, 47861, 51790, 53825
UMBC	MT00001489, MT00002218, MT00005611, MT00006795, MT00014476, MT00014477, MT00024208, MT00040458, MT00044517, MT00067127, MT00097253, MT00123405, MT00128126, MT00128193, MT00128218, MT00180255, MT00180319
UNA	UNA00057050, UNA00060828, UNA00066472
UPS	V-149629
NPGS	1064753, 1082310, 1649451, 1655096
UVSC	9816
UWBM	9614, 9615, 64325, 64384, 64411, 64414, 64423

			YPM	CBS 023670, CBS 023683, CBS 023689, YU 056356
	Literature	0		
	Survey	0		
	Observation	23	iNat	78470, 113086, 174545, 200211, 203679, 227855, 240840, 284343, 295025, 426778, 427615, 448044, 451421, 456240, 476605, 518500, 548245, 711016, 845398, 920769, 1018321, 1046465
			NG	67605920
Sources	*ACAD = Acadia University E. C. Smith Herbarium, AD = ArtDatabanken, AMNH = American Museum of Natural History, BBA_BC = Breeding Bird Atlas British Columbia, BBA_MB = Breeding Bird Atlas Manitoba, BBA_ME = Breeding Bird Atlas Maine, BBA_NY = Breeding Bird Atlas New York, BBA_ON = Breeding Bird Atlas Ontario, BBA_QC = Breeding Bird Atlas Quebec, BBS = North American Breeding Bird Survey, BLB = Borror Laboratory of Bioacoustics, CM = Carnegie Museum of Natural History, CMN = Canadian Museum of Nature, CONN = University of Connecticut George Safford Torrey Herbarium, CRCM = Charles R. Connor Museum, CSU = Colorado State University Herbarium, CUML = Cornell University Macaulay Library, eBird = eBird, EMBL = European Molecular Biology Laboratory, ESU = Emporia State University H.A. Stephens Herbarium, FLMNH = Florida Museum of Natural History, FM = Finnish Museum of Natural History, FMNH = Field Museum of Natural History, GBIF = Global Biodiversity Information Facility, iNat = iNaturalist, KU = University of Kansas Biodiversity Institute, LEA = University of Lethbridge, LGL = LGL Environmental Research Associates survey, Lit. 1 = Marshall 2001, Lit. 2 = Lewis and Starzomski 2015, Lit_3 = Ralston et al. *in review*, Lit_4 = FitzGerald et al. *accepted*, LSU = LSU Shirley C. Tucker Herbarium, MBA = Breeding Bird Atlas Maritimes, MBW = Mountain BirdWatch, MO = Missouri Botanical Garden Tropicos herbarium, MSB = Museum of Southwestern Biology, MSU = Michigan State University, MVZ = Museum of Vertebrate Zoology, UC Berkeley, NG = naturgucker.de, NMNH = National Museum of Natural History, Smithsonian Institution, NPGS = U.S. National Plant Germplasm System, NTNU = Northwest Territories/Nunavet Checklist, NYBG = New York Botanical Garden Herbarium, NYSM = New York State Museum, ORE = Oregon State University, OSUM = Ohio State University, PMAE = Provincial Museum of Alberta, Edmonton, PNMT = Parc National du Mont-Tremblant, RBCM = Royal British Columbia Museum, RBGE = Royal Botanic Garden Edinburgh, RBGK = Royal Botanic Gardens, Kew, ROM = Royal Ontario Museum, SBMNH = Santa Barbara Museum of Natural History, SNOMNH = Sam Noble Oklahoma Museum of Natural History, SNSB = Staatliche Naturwissenschaftliche Sammlungen Bayerns, SONA = Suivi des oiseaux nichant en altitude [International Bicknell's Thrush Conservation Group], UABM = University of Alberta Museums, UAM = University of Alaska Museum of the North, UAZ = University of Arizona Herbarium, UBC = University of British Columbia Herbarium, UL = Université Laval Herbier Louis-Marie, UMB = University of Manitoba Herbarium, UMBC = Université de Montréal Biodiversity Centre Marie-Victorin Herbarium, UNA = University of Alabama Biodiversity and Systematics Herbarium, UPS = Museum of Evolution in Uppsala, USU = Utah State University, UVSC = Utah Valley State College Herbarium, UWBM = University of Washington Burke Museum, VU = Vanderbilt University, WFVZ = Western Foundation of Vertebrate Zoology, XC = xeno-canto, YPM = Yale University Peabody Museum<br>^MBW survey data were obtained via eBird, and eBird catalogue numbers are listed.			

APPENDIX S2. Sources examined for tree species selection. Relative abundance averages (for Bicknell's Thrush) and importance values (for Gray-cheeked Thrush) are highlighted, and tree species often co-distributed with each thrush species are bolded.

		Abies balsamea	*Larix laricina*	***Picea glauca***	***P. mariana***	*P. rubens*	*Alnus*^	***Betula papyrifera***	*B. glandulosa*	*Prunus pensylvanica*	*Sobus americana*	*Populus balsamifera*	*P. tremuloides*	*Salix*^
Bicknell's Thrush	Morse 1979*	53.7	0	0	0	7.7	0	33.7	0	4.7	0	0	0	0
	Connolly et al. 2000	63.5	0	1.9	0	1.9	0	27.9	0	0	4.8	0	0	0
	Nixon et al. 2001	24.0	0	0	0.1	0	0	45.0	0	22.4	0.0	0	0.2	0
	Aubry et al. 2011	60.7	0	0.1	0	0.1	0	35.4	0	1.7	1.7	0	0	0
	McKinnon et al. 2014	64.9	0	0.3	0	0.3	0	22.3	0	0.5	2.7	0	0	0
	REL. AB. AVERAGE	**53.4**	0	0.4	0	2.0	0	**32.9**	0	5.9	1.8	0	0	0
	Standard deviation	17.0	0	0.8	0	3.3	0	8.5	0	9.4	2.0	0	0.1	0
Gray-cheeked Thrush	Spindler and Kessel 1980	0	3.3	17.9	32.1	0	13.5	4.6	0	0	0	7.5	0	18.6
	Kessel 1998	0	0	35.1	32.8	0	29.0	0.2	10.7	0	0	24.4	0	2.6
	FitzGerald et al. *accepted*	62.7	9.3	13.2	35.2	0	15.8	11.9	0	0.7	1.5	0.0	0	2.7
	IMPT. VAL. AVERAGE	**20.9**	4.2	**22.1**	**33.3**	0	19.5	5.6	3.4	0.2	0.5	10.6	0	8.0
	Standard deviation	36.2	4.7	11.5	1.6	0	8.3	5.9	6.2	0.4	0.9	12.5	0	9.2

* Total area was estimated

^Trees were only defined to genus

APPENDIX S3. Tree species distribution models and occurrences (white dots). Darker colors indicate areas of higher suitability. AUC values are shown. The *Picea glauca* model had a low AUC value (0.827) and was not included as a biotic factor for thrush models.

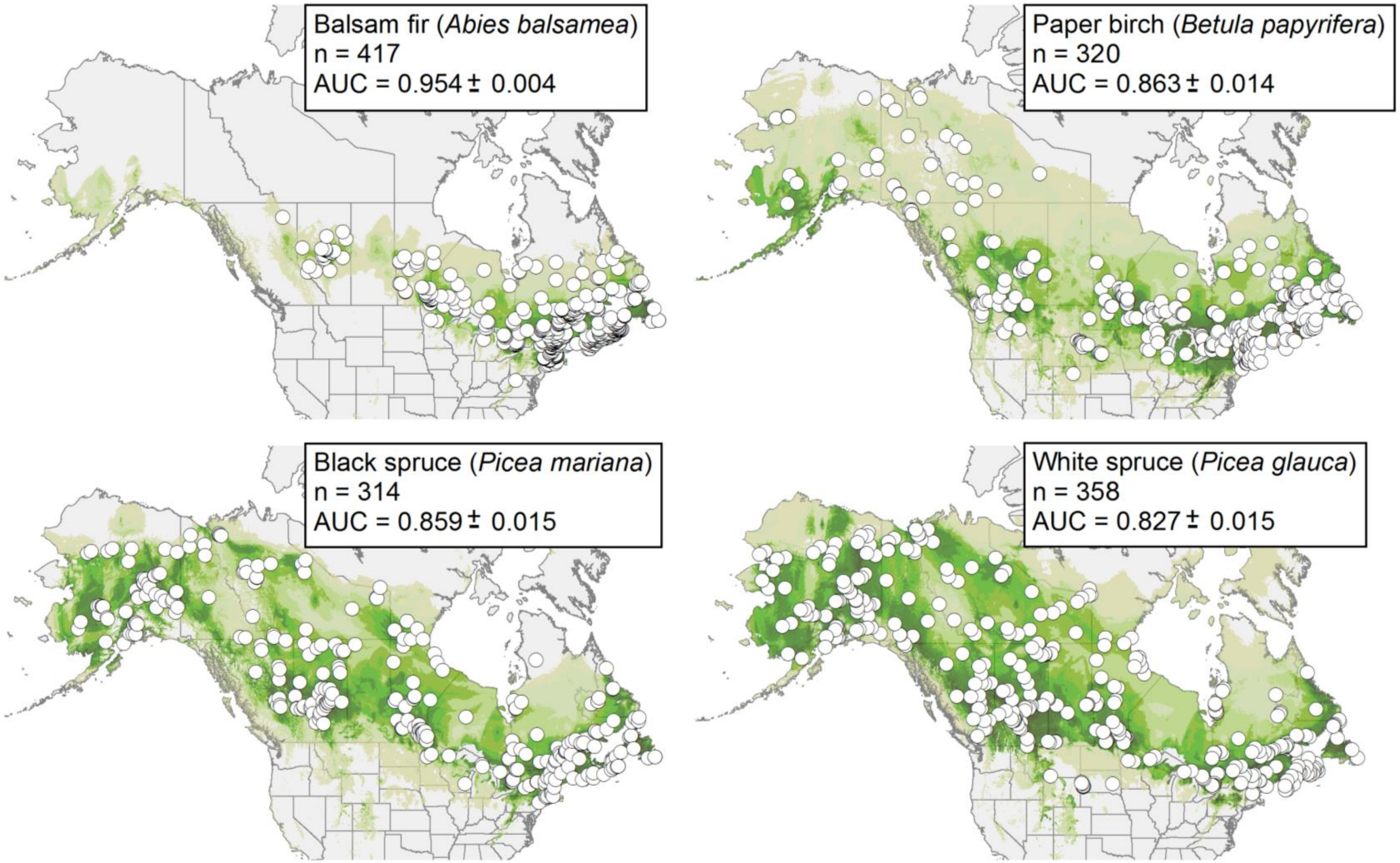

APPENDIX S4. AUC scores of ecological niche models created with different variable sets. Model (a) climate-only; (b) climate+trees; (c) climate+shapefiles; (d) trees-only. Mean AUC values (dots) and the standard deviation (bars) for each model type are shown. All models were run for 100 replicates in Maxent. Similar letters that mean AUC scores were not different ($p > 0.05$).

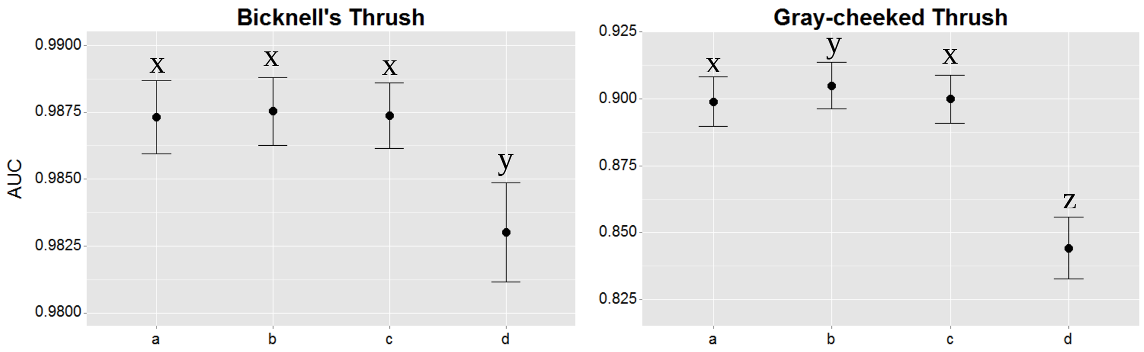

APPENDIX S5. Ecological niche models created with different variable sets. (*a*) shows Bicknell's Thrush ENM and (*b*) shows Gray-cheeked Thrush ENM. Maxent output distributions are shown using an equal sensitivity and specificity logistic threshold for comparison purposes only. Model (d) is not displayed in either figure because using trees-only greatly over-projected distributions.

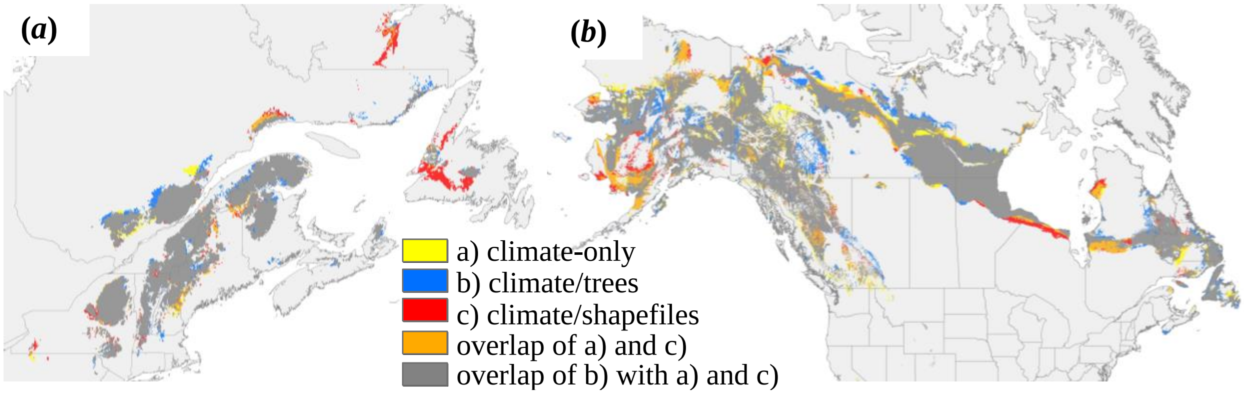

Supplement: Supplementary file 1 [file ECE3-7-5285-s001.pdf]
